# Ultrasound-guided erector spinae plane catheter versus video-assisted paravertebral catheter placement in minimally invasive thoracic surgery: comparing continuous infusion analgesic techniques on early quality of recovery, respiratory function and chronic persistent surgical pain: study protocol for a double-blinded randomised controlled trial

**DOI:** 10.1186/s13063-021-05863-9

**Published:** 2021-12-28

**Authors:** Aneurin Moorthy, Aisling Ni Eochagain, Eamon Dempsey, Donal Buggy

**Affiliations:** 1 Division of Anaesthesiology & Perioperative Medicine, Mater University Hospital, Dublin, Ireland; 2 Department of Anaesthesia and Critical Care, St James’s University Hospital, Dublin, Ireland

**Keywords:** Erector spinae catheter, Paravertebral catheter, Minimal invasive thoracic surgery, Quality of recovery, Chronic persistent surgical pain, Randomised controlled trial

## Abstract

**Background:**

Compared to conventional thoracotomy, minimally invasive thoracic surgery (MITS) can reduce postoperative pain, reduce tissue trauma and contribute to better recovery. However, it still causes significant acute postoperative pain. Truncal regional anaesthesia techniques such as paravertebral and erector spinae blocks have shown to contribute to postoperative analgesia after MITS. Satisfactory placement of an ultrasound-guided thoracic paravertebral catheter can be technically challenging compared to an ultrasound-guided erector spinae catheter. However, in MITS, an opportunity arises for directly visualised placement of a paravertebral catheter by the surgeon under thoracoscopic guidance. Alongside with thoracic epidural, a paravertebral block is considered the “gold standard” of thoracic regional analgesic techniques. To the best of our knowledge, there are no randomised controlled trials comparing surgeon-administered paravertebral catheter and anaesthesiologist-assisted erector spinae catheter for MITS in terms of patient-centred outcomes such as quality of recovery.

**Methods:**

This trial will be a prospective, double-blinded randomised controlled trial. A total of 80 eligible patients will be randomly assigned to receive either an anaesthesiologist-assisted ultrasound-guided erector spinae catheter or a surgeon-assisted video-assisted paravertebral catheter, in a 1:1 ratio following induction of general anaesthesia for minimally assisted thoracic surgery. Both groups will receive the same standardised analgesia protocol for both intra- and postoperative periods. The primary outcome is defined as Quality of Recovery (QoR-15) score between the two groups at 24 h postoperative. Secondary outcomes include assessment of chronic persistent surgical pain (CPSP) at 3 months postoperative using the Brief Pain Inventory (BPI) Short Form and Short Form McGill (SF-15) questionnaires, assessment of postoperative pulmonary function, area under the curve for Verbal Rating Score for pain at rest and on deep inspiration versus time over 48 h, total opioid consumption over 48 h, QoR-15 at 48 h, and postoperative complications and morbidity as measured by the Comprehensive Complication Index.

**Discussion:**

Despite surgical advancements in thoracic surgery, severe acute postoperative pain following MITS is still prevailing. This study will provide recommendations about the efficacy of an anaesthesia-administered ultrasound-guided erector spinae catheter or surgeon-administered, video-assisted paravertebral catheter techniques for early quality of recovery following MITS.

**Trial registration:**

ClinicalTrials.govNCT04729712. Registered on 28 January 2021. All items from the World Health Organization Trial Registration Data Set have been included.

## Administrative information

Note: the numbers in curly brackets in this protocol refer to SPIRIT checklist item numbers. The order of the items has been modified to group similar items (see http://www.equator-network.org/reporting-guidelines/spirit-2727-statement-defining-standard-protocol-items-for-clinical-trials/).

**Table:** 

Title {1}	Ultrasound guided erector spinae plane (ESP) catheter versus video-assisted paravertebral catheter placement in minimally invasive thoracic surgery (MITS): Comparing continuous infusion analgesic techniques on early quality of recovery, respiratory function and chronic persistent surgical pain: study protocol for a double-blinded randomised controlled trial**.**
Trial registration {2a and 2b}.	This trial was pre-registered on ClinicalTrials.gov Identifier: NCT04729712. Registered on 28 January 2021. All item from the World Health Organisation Trial Registration Data set have been included. https://clinicaltrials.gov/ct2/show/NCT04729712
Protocol version {3}	Protocol version 8: 01 May, 2021
Funding {4}	This trial is being funded internally from the division of Anaesthesiology & Perioperative Medicine, Mater University Hospital. The trial has also received external funding (total of 1,000 euros) from the Irish Society of Regional Anaestheia (ISRA).
Author details {5a}	(1): Dr Aneurin Moorthy*: Anaesthesia research fellow, Division of Anaesthesiology & Perioperative Medicine, Mater University Hospital, Dublin, Ireland; aneurin.moorthy@gmail.com*Correspondence (2): Dr Aisling Ni Eochagain: Anaesthesia Specialist Registrar, Department of anaesthesia and critical care, St James’s University Hospital, Dublin, Ireland; aislingnie@gmail.com. (4): Dr Eamon Dempsey: Anaesthesia Specialist Registrar, Department of anaesthesia and critical care, St James’s University Hospital, Dublin, Ireland; edempsey376@msn.com (3): Professor Donal J. Buggy**: Consultant Anaesthesiologist, Division of Anaesthesiology & Perioperative Medicine, Division of Anaesthesiology & Perioperative Medicine, Mater University Hospital, Dublin, Ireland; donal.buggy@ucd.ie **Principal investigator
Name and contact information for the trial sponsor {5b}	Division of Anaesthesiology & Perioperative Medicine, Division of Anaesthesiology & Perioperative Medicine, Mater University Hospital, Dublin, Ireland. Anaes@mater.ie office: Office: 003531803 2286/2281
Role of sponsor {5c}	This is a hypothesis-driven, investigator-initiated trial. Therefore, the funders played no role in the design of the study, data collection, analysis, interpretation of data or in the writing of the manuscript.

## Introduction

### Background and rationale {6a}

Minimally invasive thoracic surgery (MITS) is a surgical method used to perform lung surgery through small incisions between the ribs and includes both video-assisted thoracic surgery (VATS) and robotic-assisted thoracic surgery (RATS) [1]. MITS has increased to almost half of all thoracic surgery in the past decade [2]. Compared to conventional thoracotomy, MITS can reduce postoperative pain, reduce tissue trauma and contribute to better recovery [3, 4]. However, it still causes severe acute postoperative pain [3] and is a risk factor for developing chronic post-surgical pain (CPSP) [5].

Erector spinae plane (ESP) block has emerged as a new regional anaesthesia technique which has had promising early results in attenuating this severe acute pain of MITS. In a recent RCT among MITS patients, single-shot ESP block improved QoR-15 and reduced overall complications at 24 h compared with single-shot serratus anterior plane block [6].

Paravertebral block (PVB) has been widely used for analgesia after thoracic surgery for over two decades, because it reduces postoperative pain and opioid requirements compared with systemic analgesia [7, 8] and, alongside epidural analgesia, is considered the “gold standard” of thoracic regional analgesic techniques [9].

Both ESP and PVB have usually been described as a single-shot technique. This has the advantage of convenience but is limited by the finite duration of analgesia, extending to no more than 12 h at maximum and often considerably less [6, 10]. Catheter techniques offer the prospect of flexibility and prolonged analgesia.

Placement of a thoracic paravertebral catheter can be achieved by an anatomical landmark or ultrasound technique [11]. The ultrasound technique has a higher success rate and safer profile when compared to the landmark technique [12] but is technically challenging. Moreover, despite correct detection of the paravertebral space by ultrasound, the final location of paravertebral catheters can be unknown or problematic and cadaveric studies have revealed that catheters have been misplaced in up to 40% of placements [13, 14].

However, in MITS, an opportunity arises for directly visualised placement of a paravertebral catheter by the surgeon under thoracoscopic guidance [15]. In a meta-analysis [16] comparing paravertebral to epidural analgesia, lower failure rates in paravertebral placement were attributed to the practice of intraoperative surgical placement of the catheter under direct vision. Therefore, insertion of the catheter and verification of its location in the thoracic paravertebral space, by the surgeon under direct vision, could be a more reliable and safe method to guarantee drug delivery to the desired location. However, there are no randomised controlled trials examining the efficacy of this technique to ultrasound-guided paravertebral catheter insertion.

In addition, there are limited clinical effectiveness trials on the catheter-based ESP analgesia technique using patient-centred outcomes. Furthermore, no study has evaluated the effect of acute analgesia over 24–48 h with continuous regional nerve block on chronic persistent surgical pain (CPSP) at 3 months after MITS.

### Objectives {7}

Our main aim and corresponding hypotheses are outlined below.

We will conduct a randomised controlled clinical trial comparing the efficacy of anaesthesiologist-administered, ultrasound-guided ESP catheter analgesia to surgeon-administered, video-assisted PVB catheter analgesia. We will test the hypothesis that anaesthesiologist-administered ultrasound-guided ESP catheter analgesia is equivalent to surgeon-administered, video-assisted PVB catheter analgesia, in terms of early Quality of Recovery (QoR-15) and postoperative respiratory spirometric function at 24–48 h and CPSP at 3 months after MITS surgery.

### Trial design {8}

**Fig. 1:**
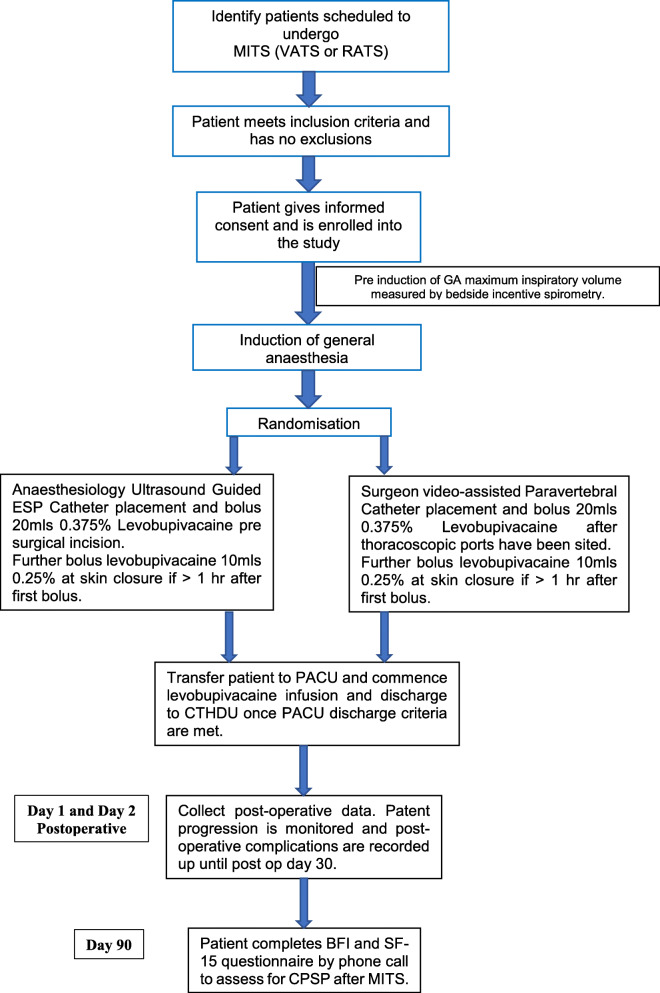
Study flow chart

This manuscript outlines a research protocol for a double-blind, randomised controlled, equivalence trial. This study is designed as equivalence, as the analgesic effectiveness of a successful paravertebral block is not questioned. Patients that volunteer to participate in this clinical trial will be equally (1:1) and randomly allocated into one of two intervention groups: anaesthesiologist-administered ultrasound-guided erector spinae catheter group (ESP catheter group) or surgeon-administered, video-assisted paravertebral block catheter group (PVB group). Figure 1 illustrates the study flow chart. Recruitment commenced on 12 May 2021, and it is expected to take between 9 and 12 months for completion.

## Methods: participants, interventions and outcomes

### Study setting {9}

The authors aim to conduct a multicentre trial. Ethical approval has been granted for Mater Misericordiae University Hospital (MMUH) and St James’s University Hospital (SJH), Dublin, Ireland, and recruitment has begun. We are currently assessing the suitability and seeking ethical approval for one other potential clinical site in Dublin, Ireland.

### Eligibility criteria {10}

The eligibility criteria for patients to enrol in this study are as follows:

Inclusion criteria:

1. ASA grade 1 to 4 patients
2. Male and female age 18–85 years
3. Unilateral MITS (VATS and RATS)

Exclusion criteria:

1. Absence of written consent
2. Unexpected conversion of MITS to open thoracotomy
3. Contraindications to peripheral regional anaesthesia block:
  - Infection at the local site
  - Allergy to local anaesthesia medications
  - Patient refusal
  - Previous or existing neurological deficit
  - Anticoagulated patients
4. Known dementia at time of MITS and inability to give informed consent
5. Unexpected postoperative admission to ICU for continued ventilation
6. Existing chronic pain condition
7. History of opioid abuse

### Who will take informed consent? {26a}

Potential participants for this trial will be identified by a member of the surgical, anaesthetic or research team. A member of the research team will analyse these patient’s electronic medical records to determine if they are potential candidates for this trial, i.e. if they meet the inclusion criteria and have no exclusions.

The suitable patient will be approached the evening before surgery if available. Alternatively, patients will be approached on the ward on the morning of surgery and their suitability to participate in the trial will be confirmed. The purpose of the trial, peripheral nerve blocks (including benefits and risks) and method of follow-up will be explained to the patient. A comprehensive and informative leaflet will be given to each patient, and they will be afforded an adequate amount of time (minimum 10 min) to study it. Participants will be informed that their participation in the study is entirely voluntary and they will have the opportunity to withdraw from the study at any time and this will not affect the quality of care they receive. Subsequently, a member of the research team will obtain informed written consent from the participant.

### Additional consent provisions for collection and use of participant data and biological specimens {26b}

Not applicable. No data and biological specimens will be collected for use in ancillary studies.

### Interventions

#### Explanation for the choice of comparators {6b}

Erector spinae block has been embraced by many anaesthesiologists; however, to the best of our knowledge, there is no clinical effectiveness trial comparing anaesthesiologist-administered, ultrasound-guided erector spinae catheter to surgeon-administered, video-assisted paravertebral block catheter techniques for minimally invasive thoracic surgery in terms of patient-centred outcomes and therefore this clinical trial is warranted.

#### Intervention description {11a}

##### ESP catheter group: anaesthesiologist-administered, ultrasound-guided erector spinae catheter

This peripheral nerve block will be performed or supervised by a consultant anaesthesiologist in regional anaesthesia with experience in the placement of an ESP catheter. The block will be performed as follows: the patient will be positioned in the lateral decubitus position, and the skin will be cleaned with chlorhexidine gluconate 0.5%/isopropyl alcohol 70% solution (ChloraPrep; Becton Dickinson, New Jersey, NJ, USA). A linear ultrasound transducer (SonoSite HFL 50x; SonoSite Inc.) will be placed in a sterile cover. This ultrasound probe will be used to identify the T5 spinous process and the erector spinae muscle group superficial to it. An 18G Tuohy Epidural needle (B. Braun medical) will then be advanced in a cranial-caudal direction. The needle tip will be slowly advanced under ultrasound guidance (in-plane technique) until it is located within the interfascial plane deep to the erector spinae muscle group and superior to the transverse process. Once in position, 10 ml of 0.9% normal saline will be injected to confirm a satisfactory needle position. Following this, 20ml 0.375% levobupivacaine will be injected and then a 20 G Nylon Epidural catheter (Perifix Epidural Catheter, B. Braun medical) will be advanced into this interfascial plane. The catheter will then be attached to a Luer lock connector with an antibacterial filter and secured to the patients back using surgical steri strips and standard dressings. A further bolus of 10ml 0.25% levobupivacaine will be administered via the catheter towards the end of the surgical procedure (> 1 h since the last dose).

##### PVB catheter group: surgeon-administered, video-assisted paravertebral block catheter

This nerve block will be performed or supervised by a consultant thoracic surgeon with experience in the placement of a paravertebral catheter under thoracoscopic guidance. The paravertebral block will be conducted at the start of the surgery after the thoracoscopic ports have been inserted. The block will be performed as follows: A percutaneous puncture point, which is equidistant to the upper and lower intercostal space, 2–3 cm lateral to the midline at the level between T4 and T5 will be marked. An 18 G Tuohy Epidural needle (B. Braun medical) will be advanced perpendicularly through the chest wall at this marked point to lie within the paravertebral space. The thoracoscope will be used to ensure that the epidural needle does not puncture the pleura and then 10–20 ml of 0.9% normal saline will be injected through the epidural needle to confirm a satisfactory position of the needle. This will appear as an induration under the pleura which can be easily seen thoracoscopically. A 20 G Nylon Epidural catheter (Perifix Epidural Catheter, B. Braun medical) will then be advanced into the paravertebral space through the epidural needle. Again, the thoracoscope will be used to ensure that the epidural catheter does not puncture the pleura and lies within the paravertebral space. The catheter will then be attached to a Luer lock connector with an antibacterial filter and secured to the patients back using surgical steri strips and standard dressings. A bolus of 20 ml 0.375% levobupivacaine will then be injected into the paravertebral space by using the catheter.

A further bolus of 10ml 0.25% levobupivacaine will be administered via the catheter towards the end of the surgical procedure (> 1 h since the last dose).

#### Criteria for discontinuing or modifying allocated interventions {11b}

Criteria for discontinuing study protocol are as follows:

1. Unexpected conversion of MITS to open thoracotomy
2. Unexpected regional anaesthesia complication
3. Suspected or confirm diagnosis of local anaesthesia toxicity
4. Unexpected postoperative admission to the intensive care unit
5. Inability to place ESP or PVB catheter
6. Patient request to be withdrawn from the study
7. Clinical concern of patient’s care from surgical, anaesthesia or research team

#### Strategies to improve adherence to interventions {11c}

**Fig. 2:**
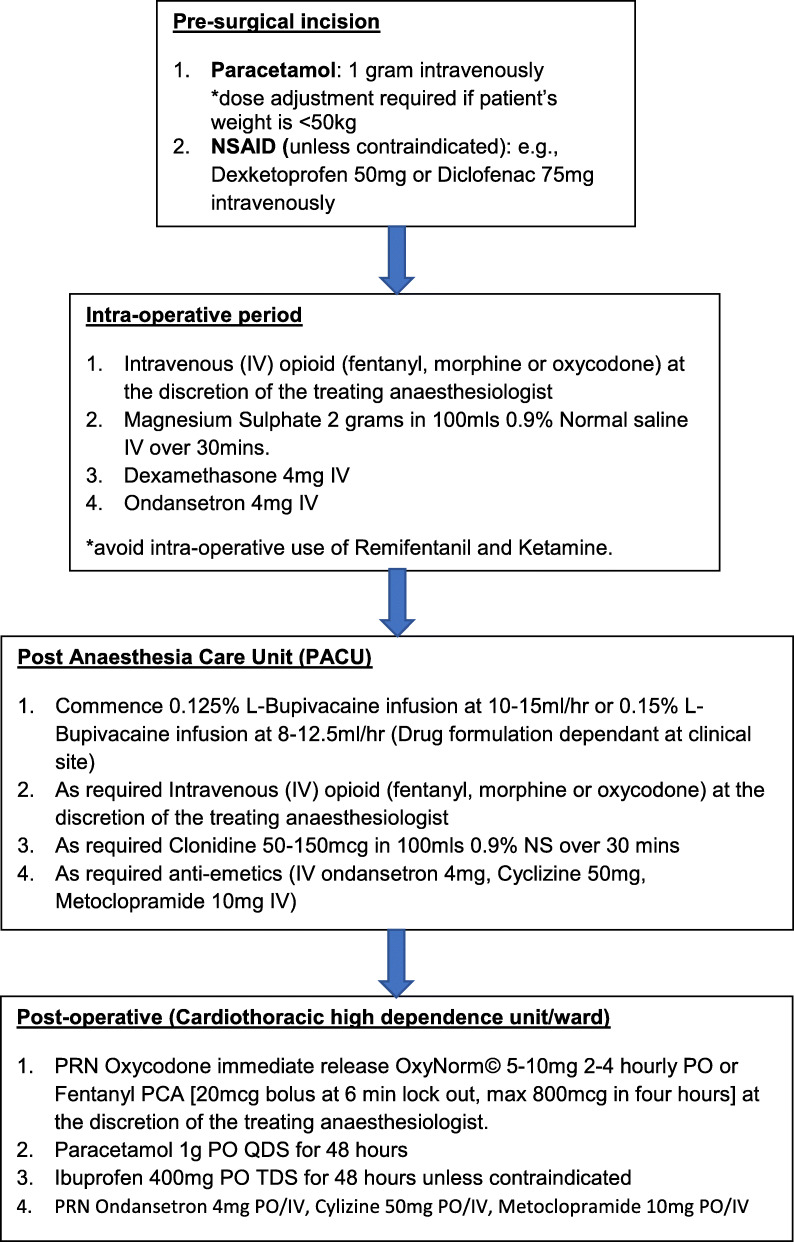
Analgesia and anti-emetic protocol

A study flow chart (Fig. 1) and analgesia and anti-emetic protocol (Fig. 2) will be made available in the operating theatre, surgeons and anaesthesiologist performing the intervention, postanaesthesia care unit (PACU) department and data collectors for the purpose of protocol adherence.

### Relevant concomitant care permitted or prohibited during the trial {11d}

All patients will undergo general anaesthesia (GA) as part of the standard of care for their video-assisted thoracic surgery. Intravenous induction of GA will be conducted or supervised by a consultant anaesthesiologist. Induction of GA will be achieved by administrating intravenous fentanyl, oropofol and a neuromuscular blockade agent at the discretion of the anaesthesiologist. An analgesia and anti-emetic concomitant care plan will be standardised for the pre-surgical, intraoperative, PACU and postoperative periods. This is outlined in Fig. 2.

### Provisions for post-trial care {30}

Patients that are enrolled into the study are covered by indemnity for negligent harm, through the standard Health Service Executive (HSE) indemnity arrangements. If any participant suffers from any complications arising directly from either intervention, he/she will receive standard postoperative management which may include from the surgical team, pain medicine department and allied health care professions. In addition, all investigators in all potential clinical sites will be respective employees of the hospital and covered by the clinical indemnity scheme.

### Outcomes {12}

#### Primary outcome

The primary outcome in this study is the Quality of Recovery (QoR-15) score between anaesthesiologist-administered ultrasound-guided erector spinae block and surgeon-administered video-assisted paravertebral block at 24 h postoperative. This score is a measure of overall postoperative recovery across five dimensions of health: patient support, comfort, emotions, physical independence and pain [17].

#### Secondary outcome measures

The following secondary outcomes will be measured:

1. Chronic persistent surgical pain (CPSP) after minimally invasive thoracic surgery (MITS) at postoperative day 90 (this will be achieved by assessing the patient for chronic pain by using the Brief Pain Inventory (BPI) Short Form and Short Form McGill (SF-15) questionnaires)
2. Pulmonary function assessment ( this will be evaluated preoperatively and at postoperative days 1 and 2 using a bedside incentive spirometry)
3. Area under the curve (AUC) of Verbal Rating Score (VRS) for pain at rest and on deep inspiration versus time over 48 h
4. Time taken to site ESP and PVB catheter
5. Time of administration of first intravenous opioid
6. Total 24- and 48-h morphine-equivalent consumption
7. Nausea, vomiting and pruritis
8. Regional anaesthesia block-related complications (e.g. block failure and catheter dislodgement)
9. Duration of postanaesthesia care unit (PACU) stay
10. Length of hospital stay and QoR-15 at 48 h
11. Summary of postoperative complications (this will be measured by calculating the Comprehensive Complication Index (CCI) at postoperative day 30)

#### Other study parameters

The following data will also be collected: patient gender, age, height, weight, American Society of Anaesthesiologist (ASA) grade and type of surgery.

### Participant timeline {13}

**Fig. 3:**
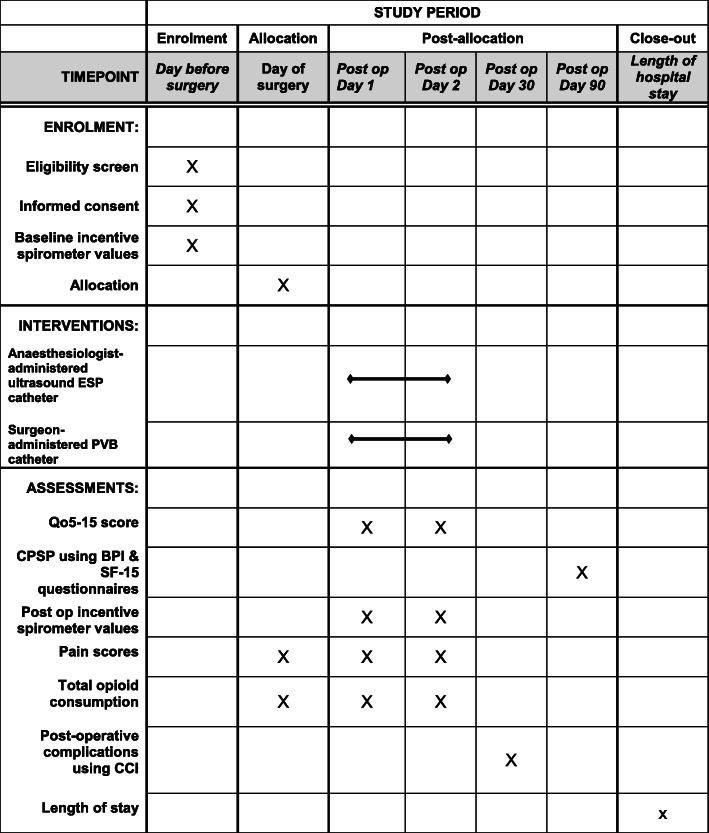
Time schedule of enrolment, interventions, assessments and visits for participants

The schedule of enrolment, interventions and assessments is outlined in Fig. 3.

### Sample size {14}

The established minimum clinically important difference (MCID) in QoR-15 score after surgery is 8.0 [18], and the *SD* of QoR-15 scores is typically between 10 and 16 [range of QoR score is 1–150]. Therefore, we considered a reduction in mean QoR-15 scores by 8 to be clinically meaningful and used this value as our delta (Δ) limit. In addition, we have chosen a standard deviation (*SD*) of 12 to reflect our study population. Based on these assumptions, we considered the following for our sample size analysis. For a two-sided test, assuming a type I error (*α*) = 0.05 and type II error (*β*) = 0.2, a sample size of *n* = 36 per group will have 80% power to detect a statistically significant difference in mean QoR-15 scores.

We aim to enrol *n* = 40 each group to allow for loss to follow-up, missing data or withdrawal of consent. The sample size was estimated using the following formula [19]: *n* = 2*σ*^2^ (*z*1 − *α*/2 + *z*1 − *β*)^2^/Δ^2^, where *α* and *β* are the probabilities of type I and II errors, *σ* is the *SD* and Δ is the delta limit. This calculation was subsequently verified by an online calculator (Georgiev G.Z., “*Sample Size Calculator*”, Available at: https://www.gigacalculator.com/calculators/power-sample-size-calculator.php).

### Recruitment {15}

Members of the anaesthestic, surgical and research team from the clinical site will participate in the recruitment process.

### Assignment of interventions: allocation

#### Sequence generation {16a}

Patients will be randomised to either the ESP or the PVB group by using an online computer-generated block randomisation. Block randomisation will occur in groups of 10 to ensure even numbers of participants in each arm of the study. The investigators for this trial will not have access to the randomisation key/seed until the study has been completed.

#### Concealment mechanism {16b}

The patient study number and group allocation will be typed onto separate pages and concealed in sequentially numbered, opaque, sealed envelopes. The randomisation process will be performed by an independent third party that is not involved in conducting this trial.

#### Implementation {16c}

After confirming the informed consent form for participating in this trial has been signed by the participant, the sealed envelope will be opened by the treating anaesthesiologist to reveal the group allocation. This process will occur *after* induction of general anaesthesia to ensure the patient is blinded to the study intervention.

### Assignment of interventions: blinding

#### Who will be blinded {17a}

This study will be a double-blinded clinical trial. Patients will be blinded to the study because they will receive the intervention after they have been put under GA. Members of the research team involved in the data collection and analysing the data will be masked to group allocation. The treating anaesthesiologist and surgical team will not be blinded

#### Procedure for unblinding if needed {17b}

A participant’s allocations will be revealed immediately if there was a clinical concern, i.e. if the patient met the criteria for discontinuing study protocol (part 11b).

### Data collection and management

#### Plans for assessment and collection of outcomes {18a}

Data collection will occur at three time points (preoperative, intraoperative and postoperative). Data will be derived from a combination of electronic and paper patient records and directly from the patient by means of completing a questionnaire. In addition, further postoperative data (assessing for CPSP) will be collected by phone call after the patient has been discharged. In each clinical site, a member of the research team, a senior anaesthesiologist, non-consultant hospital doctor, will be nominated with the primary task of data collection. In addition, he/she will be blinded to the group allocation, will not be involved in the data analysis process and will not perform the study interventions (i.e. anaesthesiologist-administered, ultrasound-guided ESP catheter insertion). Prior to patient enrolment, the designated data collectors will receive training from the principal investigator (DB), in regard to the completion process of the CRF and the various questionnaires (QoR-15, BPI, SF-15, etc.) that will be used for this study.

The primary outcome will be measured by completing the Quality of Recovery Score (QoR-15) questionnaire [20] at 24 and 48 h postoperative. QoR-15 is a 15-parameter questionnaire, and it is scored between 0 and 150, where 150 indicates that the patient has had an excellent recovery. QoR-15 has been recommended as an optimum tool to evaluate overall patient recovery after surgery [21], and this includes postoperative pain. In addition, there is high-quality evidence for good content validity, reliability and internal consistency [21].

The key secondary outcomes that will be measured in this trial include the following: chronic persistent surgical pain (CPSP), postoperative respiratory function, Verbal Rating Score (VRS) and measure of postoperative complications. CPSP will be assessed at postoperative day 90 by using the Brief Pain Inventory (BPI) form and Short McGill form (SF-15) questionnaires. The designated data collector will complete these questionnaires by telephone follow-up with the study participant. BPI assesses the pain intensity (scale 0–10) and interference of pain (scale 0–10) on quality of life. It was originally developed to assess cancer pain. However, it has been validated for its use in the assessment of chronic pain in the non-malignant pain population [22, 23]. The SF-15 questionnaire is a shorter version of the original McGill Pain Questionnaire. It consists of a pain rating index which has two subscales: sensory subscale with 11 words and affective subscale with 4 words. The SF-15 is a highly valid tool to evaluate pain in patients with and without neuropathic aetiology [24, 25]. Postoperative respiratory function will be assessed by measuring the patient’s average maximum inspiratory effort, by using a bedside incentive spirometer. This parameter will be gauged by the designated data collector at three time points. These time points include the preoperative period (prior to induction of general anaesthesia) and at postoperative days 1 and 2. Area under the curve (AUC) of Verbal Rating Score (VRS) for pain at rest and on deep inspiration versus time over 48 h postoperative will be measured. This method is an accepted approach for enhanced interpretation of pain outcomes for patient care [26]. Finally, the Comprehensive Complication Index (CCI) will be used to summarise the frequency and severity of all postoperative complications. This method of measuring surgical morbidity is more sensitive than existing morbidity endpoints and it is accepted as an applicable primary endpoint in surgical trials [27]. The data collectors for this trial will retrospectively review paper and electronic medical records up to postoperative day 30 and subsequently compute the CCI score according to the established Clavien-Dindo classification (available at www.assessurgery.com). The CCI score ranges between 0 (no complication) and 100 (death).

#### Plans to promote participant retention and complete follow-up {18b}

Trial participants will receive an extensive patient information leaflet (PIL) about the study. A member of the research team will explain this PIL, study set-up and the study interventions. The 3-month follow-up call for assessment of CPSP will be explained to the patient during the consent process. The importance of completion of this follow-up will be stressed.

#### Data management {19}

All patient data collected will be handled in accordance with European Union General Data Protection Regulations (EU 2016/679). Data will be initially collected manually and then transcribed onto Microsoft Excel. Data collected from the clinical site will be stored securely in the Department of Anaesthesiology at the site hospital, on a password-protected desktop computer stored in a locked office, such that only investigators assigned to data input, processing and analysis will have access. Data will be collected directly from source documents into the de-identified encoded paper case record form (CRF) and subsequently entered into the electronic CRF. A copy of the original hardcopy CRF will be stored within a locked cabinet/office accessible to authorised personnel only in accordance with local and national regulations.

#### Confidentiality {27}

All research data will be stored using a study identification number for each patient. An identifiable patient data page reporting the assigned patient identification code will be stored separately also in a locked cabinet/office (accessible to authorised personnel only) in order to record in-hospital outcomes and postoperative 3-month follow-up, supply missing data points and allow potential monitoring visits by National Coordinating Investigators. This data page will only be made available to members of the research team responsible for data input and the principal investigator. No patient identification details will be reported in any future publications.

#### Plans for collection, laboratory evaluation and storage of biological specimens for genetic or molecular analysis in this trial/future use {33}

Not applicable, no biological samples will be collected.

### Statistical methods

#### Statistical methods for primary and secondary outcomes {20a}

The collected raw data will be initially inspected for any errors; this includes but is not limited to double entry errors, missing data and data that was incorrectly entered. Data will be analysed by GraphPad Prism version 8 (GraphPad, Salt Lake City, UT, USA). The data will be tested for normal distribution according to the Shapiro-Wilk test. Primary and secondary outcomes will be analysed as follows: normally distributed data will be compared between the two groups using the unpaired Student *t* test and non-normal distributed data will be compared by using the Mann-Whitney *U* test. All data will be summarised as mean ± *SD* or median (25–75% range) as appropriate, and *p* value < 0.05 will be considered statistically significant.

#### Interim analyses {21b}

There are no interim analyses planned.

#### Methods for additional analyses (e.g. subgroup analyses) {20b}

There are no subgroup analyses planned.

#### Methods in analysis to handle protocol non-adherence and any statistical methods to handle missing data {20c}

The primary outcome will be assessed using an unpaired Student *t* test analysis or Mann-Whitney *U* test, depending on data distribution. Given our expectation that very few patients will be lost to follow-up during their inpatient stay and protocol adherence strategies as mentioned above, we expect missing data will be reduced to a minimum when analysing the primary outcome. If a statistical method is needed to account for missing data in the secondary outcomes (e.g. CPSP survey at 3-month follow-up), multiple imputation will be used.

#### Plans to give access to the full protocol, participant-level data and statistical code {31c}

The collated data collected by the investigators will be retained for a maximum of 5 years after analysis has been completed. We will deliver a completely de-identified data set and appropriate data upon reasonable request and in agreement with the principal investigator and data protection officer.

### Oversight and monitoring

#### Composition of the coordinating centre and trial steering committee {5d}

The trial steering committee will meet monthly to evaluate progress, address ongoing organisational and logistical issues and consider any adverse effects. There will be a research leader for the clinical site of whom will provide monthly reports to the principal investigator.

#### Composition of the data monitoring committee, its role and reporting structure {21a}

A data monitoring committee (DMC) has not been appointed for this study. A data protection impact assessment (DPIA) screening tool was completed, and it was analysed by the hospital’s data protection officer (DPO). In agreement with the DPO, this study poses a low risk to the rights and freedoms of natural persons and therefore a formal DPIA was not needed. Moreover, due to the rapid expected inclusion of participants to this trial, data collection is expected to be completed in less than 9 months and known minimal inherited risks associated with this trial, a DMC was not appointed.

### Adverse event reporting and harms {22}

Any unexpected complications that may arise from this trial will be documented and reported to the principal investigator, the surgical consultant and the relevant hospital patient safety board.

### Frequency and plans for auditing trial conduct {23}

For this trial, an initial auditing process may be conducted using a risk-based approach. This would initially involve focusing on a centre which may have the largest number of enrolment and/or lost of follow-up rates. The auditing process would include exploring datasets and analysing for accuracy, missing data, duplicate data and adhering to data protection guidelines. This process would be conducted by an independent reviewer who is not involved with the current trial (e.g. research nurse that is affiliated with the clinical site but is not involved with the current trial).

A research nurse affiliated with MMUH anaesthesiology but not involved in this trial may undertake an audit involving exploring datasets from this and the other institution. This would be precipitated by risk indices including high rates of enrolment and drop-out rates.

### Plans for communicating important protocol amendments to relevant parties (e.g. trial participants, ethical committees) {25}

We define a substantial modification of the study protocol as changes which may affect the outcome of the study or patient safety. Changes include any modification to the aims of the study, study design, the inclusion or exclusion criteria or any alternations of the study interventions (using new procedural equipment or conducting intervention which deviates original description). Any amendment will be agreed upon by the principal investigator of this trial and will seek approval by the Ethics Committee/IRB. Minor changes of the protocol include any administrative changes or alternation of the analgesia plan that do not impact patient safety or the conduct of the trial (e.g. changes to anti-emetic medications). The Ethics Committee/IRB may be notified of minor changes at the discretion of the principal investigator.

### Dissemination plans {31a}

The results from this clinical trial will be fully disclosed by means of publication in an international peer-reviewed journal and by oral/poster presentations at national and international scientific meetings. Both positive and negative results will be reported.

## Discussion

In conducting this randomised control trial, we aim to investigate the efficacy, in terms of quality of recovery, of either an anaesthesiologist-assisted ultrasound-guided erector spinae plane catheter or surgeon-assisted video-assisted paravertebral catheter, following induction of general anaesthesia for minimally assisted thoracic surgery. Alongside epidurals, paravertebral blocks have been widely used for analgesia after thoracic surgery for many years and are considered the “gold standard” of thoracic regional analgesic techniques [9]. Surgical advancements in the form of MITS result in less tissue trauma and less postoperative pain for patients undergoing thoracic surgery and so the use of less invasive regional techniques may be more appropriate for this subgroup of thoracic patients [3, 4]. The ESP block has become progressively more popular in recent times, and its use has been reported in a range of thoracic, spinal and abdominal surgeries [28–31]. Single-shot truncal regional anaesthesia techniques such as paravertebral and ESP blocks have shown to contribute to postoperative analgesia after MITS but they are restricted by the limited duration of analgesia, which extends to no more than 12 h in duration [6, 10]. Catheter techniques offer opportunities of increased flexibility and prolonged analgesia. The use of catheter techniques for MITS is not well documented, and existing pilot feasibility trials have used the traditional end points of opioid consumption and pain scores to assess its efficacy [32]. To the best of our knowledge, no trial to date has examined the impact of ESP catheters on the quality of recovery in MITS patients.

Current evidence suggests that the implementation of enhanced recovery after surgery (ERAS) programmes in thoracic surgery may lead to improvements in functional recovery, length of stay, opioid use, complications and readmissions [33]. Furthermore, because of their estimated analgesic advantages, fascial plane catheters may have a role as part of an ERAS programme and merit further investigation.

While MITS surgery is associated with less tissue trauma and lower pain scores than open thoracic surgery, there has been a heavy reliance on the use of opioids for the management of acute pain in the postoperative period [34]. Pre-emptive administration of these agents is no longer recommended [35]. Opioid use combined with poorly controlled pain scores postoperatively has been identified as risk factors associated with opioid misuse during the postoperative period [36]. Due to current public health concerns regarding addiction to prescription opioids administered postoperatively, there is a real need to direct our focus towards non-opioid-based multimodal analgesic strategies such as fascial plane blocks.

This protocol will result in the anaesthesiologist and surgeon not being blinded to the group allocation as they will be required to perform the block. However, the primary outcome is the QoR-15 score at 24 h postoperatively which will be measured by researchers blinded to the group allocation. We have chosen to exclude patients with existing chronic pain conditions or with a history of opioid abuse. The justification for this decision comes from the fact that these patients are more likely to develop postoperative chronic pain and so the results of our 3-month chronic pain scores may be affected by the inclusion of these patients. Pain scores and quality of recovery will be recorded up to 48 h postoperatively. We acknowledge that for some patients having MITS their recovery will continue beyond 48 h; however, the acute postoperative pain following MITS is reduced by day 3 and many of our patients are discharged home at this stage. Length of stay and postoperative complications will continue to be observed after this timeframe and patients will be followed up until 3 months postoperatively. Paravertebral and ESP catheters will be placed under general anaesthesia; therefore, formal dermatomal assessment of block function will not be undertaken. Therefore, we will not be formally testing block effectiveness. However, the practice of placing these catheters under ultrasound guidance or thorascopic vision after induction of general anaesthesia is in line with common clinical practice, and therefore, our findings should still be applicable to widespread clinical practice. Patients’ preoperative QoR-15 will not be assessed in this study. Therefore, we will not have a baseline from which to compare postoperative QoR-15 scores. However, QoR-15 was designed specifically for postoperative use, and we will apply this scoring tool to both randomised cohorts equally. Moreover, the ability of QoR-15 in the immediate preoperative period to give an accurate baseline has been questioned [37]. By focusing on patient-centred outcomes in our trial design, we hope to elucidate whether these relatively straightforward fascial plane blocks can enhance recovery and improve safety for patients by reducing postoperative morbidity and complications.

### Trial status

The trial is registered on ClinicalTrials.gov identifier: NCT04729712. The current protocol is version 8 of 01/05/2021. Participant recruitment began on 12/05/2021, and full patient recruitment is estimated to be completed by March 2022.

## Abbreviations

MITS: Minimally invasive thoracic surgery
QoR-15: Quality of Recovery
CPSP: Chronic persistent surgical pain
BPI: Brief Pain Inventory
SF-15: Short Form McGill
VATS: Video-assisted thoracic surgery
RATS: Robotic-assisted thoracic surgery
ESP: Erector spinae plane
RCT: Randomised control trial
PVB: Paravertebral block
MMUH: Mater Misericordiae University Hospital
SJH: St James University Hospital
ASA: American Society of Anaesthesiology
ICU: Intensive care unit
PACU: Postanaesthesia care unit
GA: General anaesthesia
HSE: Health Service Executive
AUC: Area under the curve
CCI: Comprehensive Complication Index Calculator
*SD*: Standard deviation
PIL: Patient information leaflet
CRF: Case record form
DMC: Data monitoring committee
DPIA: Data protection impact assessment
DPO: Data protection officer
